# Integration of MicroRNA, mRNA, and Protein Expression Data for the Identification of Cancer-Related MicroRNAs

**DOI:** 10.1371/journal.pone.0168412

**Published:** 2017-01-05

**Authors:** Jiyoun Seo, Daeyong Jin, Chan-Hun Choi, Hyunju Lee

**Affiliations:** 1 School of Electrical Engineering and Computer Science, Gwangju Institute of Science and Technology, Gwanjgu, Republic of Korea; 2 College of Korean Medicine, Dongshin University, Naju-si, Jeollanam-do, Republic of Korea; Dokuz Eylul Universitesi, TURKEY

## Abstract

MicroRNAs (miRNAs) are responsible for the regulation of target genes involved in various biological processes, and may play oncogenic or tumor suppressive roles. Many studies have investigated the relationships between miRNAs and their target genes, using mRNA and miRNA expression data. However, mRNA expression levels do not necessarily represent the exact gene expression profiles, since protein translation may be regulated in several different ways. Despite this, large-scale protein expression data have been integrated rarely when predicting gene-miRNA relationships. This study explores two approaches for the investigation of gene-miRNA relationships by integrating mRNA expression and protein expression data. First, miRNAs were ranked according to their effects on cancer development. We calculated influence scores for each miRNA, based on the number of significant mRNA-miRNA and protein-miRNA correlations. Furthermore, we constructed modules containing mRNAs, proteins, and miRNAs, in which these three molecular types are highly correlated. The regulatory interactions between miRNA and genes in these modules have been validated based on the direct regulations, indirect regulations, and co-regulations through transcription factors. We applied our approaches to glioblastomas (GBMs), ranked miRNAs depending on their effects on GBM, and obtained 52 GBM-related modules. Compared with the miRNA rankings and modules constructed using only mRNA expression data, the rankings and modules constructed using mRNA and protein expression data were shown to have better performance. Additionally, we experimentally verified that miR-504, highly ranked and included in the identified modules, plays a suppressive role in GBM development. We demonstrated that the integration of both expression profiles allows a more precise analysis of gene-miRNA interactions and the identification of a higher number of cancer-related miRNAs and regulatory mechanisms.

## Introduction

MicroRNAs (miRNAs) are small non-coding RNAs, 20–24 nucleotides long, which can suppress target gene expression post-transcriptionally by recognizing the complementary target sites in the 3’ untranslated region (3’-UTR) of mRNAs [1]. MiRNAs perfectly or partially complement target mRNA sequences, leading to mRNA degradation or the suppression of translation [2]. Furthermore, the relationships between miRNAs and the target genes are complex, since multiple miRNAs target multiple mRNAs [3, 4]. MiRNAs regulate mRNAs in diverse biological pathways, and therefore, miRNA alterations may have consequences on a number of cellular processes during cancer development and progression: cell apoptosis, proliferation, cell cycle, migration, and metabolism [5]. The importance of miRNAs for cancer development and progression has been demonstrated. The elucidation of their oncogenic or tumor suppressive functions and the identification of miRNAs that may represent potential targets for cancer therapy are, therefore, crucial tasks.

Integrated miRNA and related gene analyses in different types of cancers have been the focus of many studies [6–12]. To identify potential interactions between miRNAs and genes and pathways involved in cancer development, many studies used large-scale miRNA and mRNA expression profile datasets [8–12]. Peng *et al*. [8] proposed a biclique-based approach to the construction of miRNA and mRNA modules using their expression profiles. However, only one or two miRNAs were included in each module, which is not enough to explain complex relationships between miRNAs and genes. Jin and Lee [12] constructed modules demonstrating the interactions between multiple miRNAs and mRNAs based on bi-clustering approach and a Gaussian Bayesian network. Although this approach increased our understanding of miRNA-gene relationships, mRNA expression profiles alone may not be sufficient to represent protein translation processes, involving several regulatory steps [13–15]. Therefore, the determination of relationships between genes and miRNAs using only mRNA expression data is limited.

Protein expression profiles were investigated in several studies analyzing the interactions between miRNAs and genes [16–20]. However, most of these studies explored the relationships of only a small number of specific proteins [16–19], and a small number of studies included large-scale protein expression datasets. Aure *et al*. [20] used large-scale protein expression datasets and miRNA expression profiles to demonstrate potential protein-miRNA interactions in breast cancer. However, protein and miRNA expression levels were separately clustered, and miRNA and gene expression data were not grouped together, preventing the identification of complex relationships between these molecules.

In this study, we propose two approaches to the integration of mRNA, miRNA, and protein expression data, in order to identify cancer-related miRNAs and investigate relationships between miRNAs and the regulatory networks in cancer. We present a new computational method for the ranking of cancer-related miRNAs based on the number of identified correlated genes, using both mRNA and protein datasets. Ranking lists constructed for each miRNA may advance our understanding of the cancer-related miRNAs. Additionally, we present a method for the construction of modules containing mRNAs, miRNAs, and proteins. The modules were constructed based on the SAMBA bi-clustering algorithm [21] and a Bayesian network model [22]. To construct these modules, we extended the approach proposed by Jin and Lee [12] by adding a step in which the proteins are included into mRNA-sample modules prior to the inclusion of miRNAs. The identified modules represent subgroups of highly correlated mRNAs, miRNAs, and proteins, and may explain regulatory networks between miRNAs and genes.

We applied our approaches to the study of glioblastomas (GBMs). We ranked the miRNAs related to GBM and constructed the modules containing miRNAs, mRNAs, and proteins involved in GBM-related pathways. We validated the relevance of the ranked miRNAs to GBM and examined the regulatory relationships in these modules.

## Materials and Methods

### Materials

#### GBM datasets

We used mRNA, miRNA, and protein expression datasets of GBM obtained from The Cancer Genome Atlas (TCGA) [23]. mRNA expression datasets containing 192 tumor samples and 10 unmatched normal samples, miRNA expression data, containing 192 tumor samples and 10 unmatched normal samples, and protein expression datasets containing 192 tumor samples were included in this study. Expression profiles were obtained using Agilent 244K Custom Gene Expression G4502A-07-2, Agilent 8 x 15K Human miRNA-specific microarray, and M.D. Anderson Reverse Phase Protein Array Core. From these datasets, the information about 17814 mRNAs, 470 miRNAs, and 203 proteins was collected. Out of 203 proteins, 34% (70/203) represent cancer-related genes, according to the allOnco database [24–26] and 10% (21/203) represent transcription factors, according to Transfac Public v7.0 [27] and TransmiR v1.2 [28] databases. We obtained the information about 92 GBM-related miRNAs from the Human miRNA & Disease Database v2.0 (HMDD v2.0) [29].

#### Cell proliferation and miRNA expression profiling

Human GBM cell line T98G was purchased (Korean Cell Line Bank, South Korea) and maintained in Minimum Essential Medium (MEM; Lonza, USA) with 10% fetal calf serum (FCS; Gibco, USA) and 1% antibiotic/antimycotic reagent (Sigma-Aldrich, USA). Cells were incubated in the atmosphere containing 5% CO_2_ at 37°C. The cells were detached using trypsin-EDTA (Sigma-Aldrich, USA), centrifuged (Vision Scientific, South Korea), and plated in 24-well plates (5×10^4^ cells/well). Using G-fectin, RNAi transfection reagent (Genolution, South Korea), we transfected the plated cells with hsa-miR-504-5p mimics or hsa-miR-504-5p inhibitors (Genolution, Korea). The transfected cells were further incubated for 48 or 96 h.

MiRNA expression in the transfected cells at 48 and 96 h was investigated using reverse transcription polymerase chain reaction (RT-PCR). MicroRNA First-Strand Synthesis and miRNA Quantitation kits (Takara, USA) were used. Total RNA from the transfected cells was isolated using TRIzol reagent (Gibco-BRL, USA) and the extracted RNAs were quantified using Biophotometer (Eppendorf, Germany) at 260 nm. MiRNA expression levels were determined using Gel Documentation System by AlphaEaseFC (Alpha lnnotech, USA). Additionally, miRNA expression levels in the transfected cells were also determined by quantitative PCR (qPCR) assay using qPCR kits (Geno-qPCR Kit; Genolution, South Korea). The proliferation rates of the transfected cells at 48 and 96 h were observed by EZ-cytox and methyl thiazolyl tetrazolium (MTT) assays. We performed Ez-cytox assay with 50 μL of Ez-cytox (Dogenbio, South Korea), measuring cell proliferation rates with microplate reader (Bio-Rad, USA) at 450 nm. To perform MTT assay, we added 50 μL of MTT reagent (Amresco, USA) to the cells for 4 h. We then added 400ul of DMSO (Amresco, USA) and shacked for 10 min. The cell proliferation rates were measured by microplate reader (Bio-Rad, USA) at 450 nm. The sequence of the forward primer of hsa-miR-504-5p used in this study was 5′-ACCCTGGTCTGCACTCTATC-3′ and that of the reverse primer was the universal mRQ 3′ primer (Takara, USA). The small nuclear RNA (snRNA) U6 was used for a housekeeping gene and the sequences of the forward primer and the reverse primer were 5′-GGGCAGGAAGAGGGCCTAT-3′ and 5′-AAAAATATGGAACGCTTCACGAATTTG-3′, respectively.

### Cancer-related miRNA rankings

**Fig 1:**
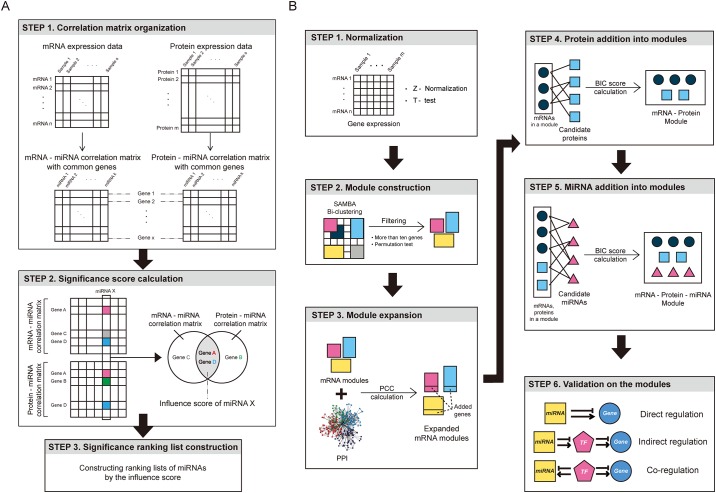
Cancer-related miRNA ranking and the construction of miRNA, mRNA, and protein modules. (A) Cancer-related miRNA ranking steps. In the first step, correlation matrices between mRNA and miRNA and between protein and miRNA expression levels are calculated. In the second step, the influence score of each miRNA is calculated using the number of genes with significant absolute correlation values with the miRNAs from both matrices. In the third step, miRNAs are ranked by influence scores. (B) The construction and the validation of three-factor modules containing miRNAs, mRNAs, and proteins expression profiles. mRNA expression matrix was normalized by Z-scores, and mRNAs shown to be differentially expressed between tumor and normal samples were selected. Next, mRNAs and samples were clustered. Following this, using protein-protein interaction data, the modules were expanded. In the fourth step, candidate proteins, showing high correlation with mRNAs in the modules, were added, followed by the addition of candidate miRNAs that are highly correlated with mRNAs and proteins. Finally, the three-factor modules were validated.

MiRNAs were ranked according to their relevance to cancer based on three steps: the building of the correlation matrices, the determination of significantly correlated gene-miRNA pairs, and the rankings of miRNAs (Fig 1A). First, we normalized the expression values of each mRNA using the Z-score. mRNAs showing significantly different expression levels between tumor and normal samples were identified using *t*-test (Excel; Microsoft, Redmond, Wash) with Bonferroni corrected *p*-values less than 0.05. From these differentially expressed mRNAs, we selected common genes included in both mRNA and protein expression datasets. To select common genes from two datasets, we mapped protein names into gene symbols. Afterward, we calculated two correlation matrices using Spearman’s rank correlation coefficients (SCCs): miRNA-mRNA expression correlation matrix and miRNA-protein expression matrix. SCCs were calculated using the cor() function found in the stats R package with a *spearman* option. In the second step, we selected significantly correlated gene-miRNA pairs in both correlation matrices. To investigate the significantly correlated pairs, top *α*% absolute correlation values in both matrices were calculated to determine the thresholds. In the final step, for each miRNA, the number of genes significantly correlated with the given miRNA in both mRNA and protein expression level datasets was determined and this number was designated as the influence score. The miRNAs were ranked in descending order based on these scores.

### The construction of mRNA, protein, and miRNA modules

The construction of modules in this study was based in part on a report by Jin and Lee [12], including four main sequential steps: the normalization of gene expression data, construction of mRNA-sample modules, the expansion of modules with additional correlated genes, and the addition of highly correlated miRNAs into modules (Fig 1B). The difference in our model is the integration of protein information, before the addition of miRNAs, to the modules.

We first normalized the expression levels of each mRNA and selected mRNAs that are differentially expressed between tumor and normal samples. Afterward, we constructed mRNA modules by applying SAMBA bi-clustering algorithm. As Jin and Lee [12] demonstrated, bi-clustering algorithm permits the duplication of the elements in the clusters and allows clusters to share genes with multiple functions. We then filtered out modules containing less than 10 mRNAs. The statistical significance of the modules obtained by bi-clustering was confirmed by comparing them with randomly generated modules. For each observed module, we constructed 1,000 random modules by randomly selecting the same numbers of genes and samples from the normalized gene expression matrix. For a random module *i*, we calculated Pearson correlation coefficients (PCC) for all gene pairs in the random module and averaged the PCC values, generating *random*_*avg*_(*i*). Additionally, we calculated the average PCC for the observed module, *observed*_*avg*_. Afterward, *p*-value for the observed module was calculated as $\sum_{i=1}^{1,000}I\left(random_{avg}\left(i\right)>observed_{avg}\right)/N$, where *I* is an indicator function. Modules having *p*-value ≤ 0.05 were selected. PCCs were calculated using the function cor() of the stats R package with a *pearson* option.

Following the previous steps, we expanded these modules by adding genes that highly interact with mRNAs in the modules. Candidate genes were selected from the protein-protein interaction (PPI) data obtained from Human Protein Reference Database (HPRD) [30]. For each candidate gene, we calculated the average PCC between the expression of the candidate gene and mRNAs in the module. Starting with the gene with the highest PCC, the candidate genes were added to the module until the average PCC of the expanded matrix stopped increasing.

In the fourth step, we added proteins to the modules. In order to select the candidate proteins, we calculated the average of absolute SCCs between the expressions of mRNAs in a module and each protein expression level. We selected the proteins with the average SCC values within the top *β* %. Bayesian network model was applied, where a joint distribution between mRNAs and proteins was calculated as the conditional probability of mRNA given its candidate parent proteins. We added the candidate proteins into the modules, starting with the protein with the highest SCC average value, and calculated the Bayesian information criterion (BIC) score of the modules at each inclusion, until this score stopped increasing. The Bayesian network and BIC score were determined using the bnlearn R package [31]. For more details about the Bayesian network model, refer to Eqs (2) and (3) in [12].

Finally, after the construction of the described modules, miRNAs were included using Bayesian network model as well. We selected candidate miRNAs shown to be significantly correlated with mRNAs and proteins in each module. For each miRNA, we calculated the average SCC value for miRNA-gene (mRNA and protein) expressions. Candidate miRNAs with the average SCC value within the top *γ*% were selected for each module. We added the candidate miRNAs to the modules, starting with the miRNA with the highest average SCC value, until the BIC score of the module no longer increased. We designated the final modules as three-factor modules, since they contain the information about three types of molecules in each module: mRNAs, proteins, and miRNAs.

### Validation of highly ranked miRNAs

Three approaches were used in order to validate whether highly ranked miRNAs in our model are related to the GBM development and progression. We obtained the information about miRNAs that are known to be related to GBM from HMDD, and compared these miRNAs with the miRNAs identified using our approach. The proportion of the previously known miRNAs was calculated for each ranking. Additionally, for a given ranking used as the threshold, we defined miRNAs that are ranked higher than the threshold ranking and are known to be related to GBM in HMDD as true positives. MiRNAs ranked higher than the threshold, but not known to be related to GBM were defined as false positives, while the ones ranked lower than the threshold and not known to be related to GBM were defined as false negatives. We constructed receiver operating characteristic (ROC) curves of true positive and false positive rates, and then calculated the area under ROC curve (AUC). Furthermore, a survival analysis was performed to validate whether the expression levels of highly ranked miRNAs significantly affect the survival time of GBM patients. Clinical data, including survival information, were obtained from TCGA. Patients were divided into two subgroups based on the expression levels of the miRNAs: a subgroup containing patients with miRNA expression levels in the top X%, and another subgroup containing patients with miRNA expression levels in the bottom X%. The mean survival times were compared using Kaplan-Maier survival analysis.

### Validation of three-factor modules

We performed an enrichment test to validate the functional relevance of mRNAs and proteins in the modules. A pathway enrichment test was performed using gene ontology (GO) [32], Kyoto Encyclopedia of Genes and Genomes (KEGG) [33], and BioCarta biological process database (http://cgap.nci.nih.gov/Pathways/BioCarta_Pathways). Hypergeometric test was applied to each module and *q*-values were computed from *p*-values using Benjamini & Hochberg correction.

To confirm the associations between mRNAs, proteins, and miRNAs in our modules, three validation steps were performed (Fig 1B). Initially, we investigated whether miRNAs in the modules directly target the genes included in the same modules. We obtained the experimentally validated target genes for each miRNA from miRTarbase [34], which contains the information about positive or negative regulatory effects of miRNAs. Even though it has been demonstrated that miRNAs generally repress genes, several recent studies revealed that the target gene expression can be upregulated by miRNAs associated with protein complexes [35–38]. For each miRNA included in the module, we applied hypergeometric test to determine common genes between the target genes obtained from miRTarbase and genes included in the same module with the given miRNA. Additionally, we investigated whether miRNAs indirectly regulate the genes in the same modules via transcription factors (TFs). For each miRNA in the modules, we constructed a list of experimentally validated target TFs using miRTarbase, and obtained the information about the experimentally validated target genes of these TFs from Transfac. For each miRNA in the modules, we compared the genes identified as indirectly regulated by the miRNAs via TFs and the genes included in the same module. By applying hypergeometric tests for significance of overlaps, *q*-values were obtained. Furthermore, we validated whether miRNAs and genes in the same module are co-regulated by TFs. For each miRNA, we obtained experimentally validated TF data, targeting the miRNA, from TransmiR. We identified the genes regulated by these TFs using Transfac. For each miRNA, we identified common genes found in these datasets and in our modules. We applied hypergeometric tests to determine the significance of overlaps, and *q*-values were obtained. Source codes for implementing our proposed methods are available at http://gcancer.org/IMMP/.

## Results

### Comparison of mRNA and protein expression levels

**Fig 2:**
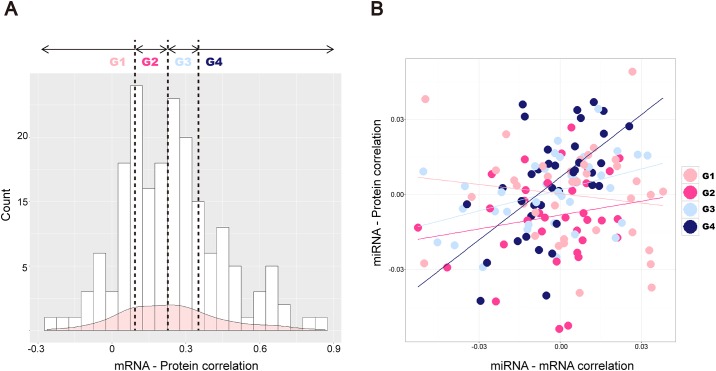
Comparison of mRNA and protein expression levels. (A) Correlations between mRNA-protein expression levels are presented. On the *x*-axis, correlation coefficients for 146 common genes are shown, while the *y*-axis presents the frequency of SCCs. Vertical lines present 25*^th^*, 50*^th^*, and 75*^th^* percentiles of the SCCs. (B) On the *x*-axis, correlation coefficients between mRNAs and miRNAs are presented, and on the *y*-axis correlation coefficients between mRNAs and proteins are presented. Each dot represents one gene, and light pink, pink, light blue, and blue colored dots represent genes in G1, G2, G3, and G4 groups, respectively. Regression lines show relationships between mRNA-miRNA correlations and protein-miRNA correlations.

We compared mRNA and protein expression levels by determining the correlations between them. We calculated SCCs of mRNA and protein expressions of 146 common genes. Interestingly, the average SCC of these genes was determined to be 0.243, which suggests differential mRNA and protein expression levels, although they are positively correlated. In Fig 2A, a histogram of SCCs calculated for the common genes is presented, and most SCCs were determined to be below 0.5. The genes were classified into four groups using the thresholds at 25*^th^*, 50*^th^*, and 75*^th^* percentiles of SCCs, corresponding to 0.090, 0.230, and 0.348, respectively. SCCs of the first group (G1) were lower than the 25*^th^* percentile, of the second group (G2) between 25*^th^* and 50*^th^* percentile, while SCCs of the third group (G3) were between 50*^th^* and 75*^th^*, and the fourth group (G4) over 75*^th^* percentile.

We then compared the correlations between these four gene groups and miRNAs. For each gene, the average SCCs of mRNA-miRNA and protein-miRNA expression levels are presented in Fig 2B. Regression lines showing the relationships between mRNA-miRNA and protein-miRNA correlations demonstrated that gene groups with higher mRNA-protein expression SCC values show more similar correlation with miRNAs, which can be observed for group G4. In contrast to this, genes belonging to G1 group, with the largest differences in mRNA and protein expression levels, have less similar SCCs between mRNA-miRNA and protein-miRNA expression levels.

### Ranking of GBM-related miRNAs

#### Ranking of miRNAs significantly associated with GBM

We collected the information about 5890 mRNAs differentially expressed in GBM patients in comparison with the normal samples, and selected 146 genes for which the protein data can be found. For every gene-miRNA expression pair, absolute PCCs (APCCs) were obtained from mRNA-miRNA and protein-miRNA expression correlation matrices, and an influence score for each miRNA was calculated based on the number of significantly correlated genes in top 1% or 5% APCCs from the two matrices. Four hundred and seventy miRNAs were ranked by their influence scores, and two ranking lists, for genes with 1% APCCs (designated as 1% GBA) or 5% APCCs (5% GBA), were constructed. Table 1 shows top 10% (47/470) of miRNAs from the two rankings, sorted by the influence scores. In S1 Table, the rankings of all miRNAs are presented.

**Table 1:** miRNA ranking. Rankings of miRNAs according to 5% ACC and 1% ACC values are shown. MiRNAs are marked with O (not included in HMDD) or X (included in HMDD) in the third and fifth columns.

Ranking	5% ACC		1% ACC	
	miRNA	HMDD included	miRNA	HMDD included
1	hsa-miR-21	O	hsa-miR-21	O
2	hsa-miR-223	X	hsa-miR-34a	O
3	hsa-miR-222	O	hsa-miR-22	O
4	hsa-miR-34a	O	hsa-miR-155	O
5	hsa-miR-130b	X	hsa-miR-210	O
6	hsa-miR-22	O	hsa-miR-29c	O
7	hsa-miR-128a	X	hsa-miR-130b	X
8	hsa-miR-155	O	hsa-miR-106b	X
9	hsa-miR-214	X	hsa-miR-199a*	X
10	hsa-miR-128b	X	hsa-miR-204	X
11	hsa-miR-210	O	hsa-miR-222	O
12	hsa-miR-221	O	hsa-miR-223	X
13	hsa-miR-29b	X	hsa-miR-34b	X
14	hsa-miR-34b	X	hsa-miR-128b	X
15	hsa-miR-9*	O	hsa-miR-137	O
16	hsa-miR-92	X	hsa-miR-142-5p	O
17	hsa-miR-17-5p	O	hsa-miR-17-3p	O
18	hsa-miR-29a	O	hsa-miR-18a	O
19	hsa-miR-199a	X	hsa-miR-19b	O
20	hsa-miR-199a*	X	hsa-miR-214	X
21	hsa-miR-19a	O	hsa-miR-29a	O
22	hsa-miR-204	X	hsa-miR-29b	X
23	hsa-miR-27a	X	hsa-miR-92	X
24	hsa-miR-29c	O	hsa-miR-504	O
25	hsa-miR-146b	O	hsa-miR-128a	X
26	hsa-miR-181d	O	hsa-miR-129	X
27	hsa-miR-18a	O	hsa-miR-15b	X
28	hsa-miR-19b	O	hsa-miR-17-5p	O
29	hsa-miR-23a	X	hsa-miR-181d	O
30	hsa-miR-30a-5p	O	hsa-miR-193a	O
31	hsa-miR-338	X	hsa-miR-199a	X
32	hsa-miR-93	X	hsa-miR-19a	O
33	hsa-miR-106b	X	hsa-miR-25	O
34	hsa-miR-142-3p	O	hsa-miR-27a	X
35	hsa-miR-142-5p	O	hsa-miR-30c	O
36	hsa-miR-17-3p	O	hsa-miR-33	X
37	hsa-miR-181a*	O	hsa-miR-124a	X
38	hsa-miR-181c	O	hsa-miR-93	X
39	hsa-miR-30a-3p	O	hsa-miR-101	O
40	hsa-miR-33	X	hsa-miR-106a	O
41	hsa-miR-488	X	hsa-miR-221	O
42	hsa-miR-99a	O	hsa-miR-139	O
43	hsa-miR-504	O	hsa-miR-30a-5p	O
44	hsa-miR-139	O	hsa-miR-181c	O
45	hsa-miR-25	O	hsa-miR-195	O
46	hsa-miR-342	O	hsa-miR-9*	O
47	hsa-miR-20a	O	hsa-miR-200c	X

miR-21, miR-34a, miR-22, miR-155, and miR-210 were shown to represent the top five significantly ranked miRNAs in 5% GBA ranking list. Top five significant miRNAs in the 1% GBA ranking list were miR-21, miR-223, miR-222, miR-34a, and miR-130b. miR-21 was commonly ranked as a miRNA with the most significant association with GBM, and it is generally overexpressed in these tumors [39, 40]. MiR-155, ranked fourth and eighth in 1% GBA and 5% GBA ranking lists, respectively, was previously reported to be associated with GBM [41, 42]. Other highly ranked miRNAs, miR-34a and miR-22, were reported as tumor suppressors, with the decreased expression levels in GBMs [43, 44].

#### Validation of the miRNA rankings

**Fig 3:**
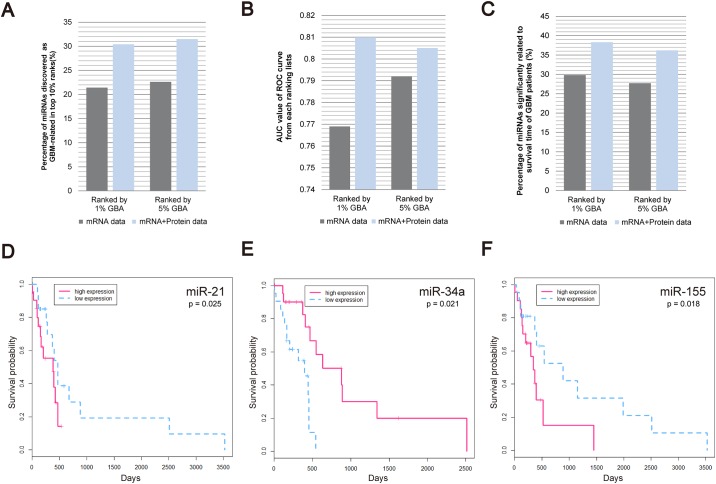
miRNA ranking list validation. (A-C): Comparison between miRNA rankings constructed using mRNA expression data (gray), and those constructed using both mRNA and protein expression data (blue). (A) The percentage of miRNAs known to be associated with GBM within the top 10% of the 470 ranked miRNAs. (B) AUC values of the ROC curves of ranking lists. (C) For miRNAs ranked in the top 10%, the percentages of miRNAs significantly associated with the changes in the survival time of GBM patients (*p*-value < 0.05) are shown. (D-F) Survival time analysis of GBM patients with high (pink dotted line) or low (blue solid line) expression levels of miR-21, miR-34a, and miR-155.

Out of all 470 ranked miRNAs, 92 were previously reported to be associated with GBM. We showed that 30.4% (28/92) and 31.5% (29/92) of the previously known miRNAs are included in top 10% of miRNAs in 1% GBA and 5% GBA ranking lists, respectively (Fig 3A). Moreover, we compared the performance of our approach with the ranking lists constructed without protein expression data. These ranking lists included a smaller number of previously known miRNAs in their top 10% rankings, with 21.7% (20/92) and 22.8% (21/92) of miRNAs included in 1% GBA and 5% GBA ranking lists, respectively (S2 Table). To further validate miRNA rankings, we assessed AUC values of ROC curves from 1% GBA or 5% GBA rankings. As shown in Fig 3B, our ranking lists, constructed using both protein and mRNA expression data, are more accurate, with the AUC values of 0.810 and 0.805, respectively, compared with the lists constructed without protein expression data (AUC values of 0.769 and 0.792 for 1% GBA and 5% GBA rankings, respectively).

In addition, we investigated whether the top 10% miRNAs are associated with the survival time of GBM patients. For each miRNA, 209 patients were divided into two groups, with high or low miRNA expression levels, and their effects on the survival time of the patients were assessed. We demonstrated that for 38.3% (18/47) and 36.2% (17/47) of miRNAs included in 1% GBA and 5% GBA ranking lists constructed with both mRNA and protein expression data, a significant association (*p* < 0.05) with the survival time was found. However, using the 5% GBA and 1% GBA rankings constructed with mRNA expression data only, 29.8% (14/47) and 27.7% (13/47) of the top 10% miRNAs were shown to be related to the survival time, respectively (Fig 3C).

In Fig 3D–3F, the survival curves of two groups of patients with high or low expression levels of specific miRNAs are presented. It was shown that the mean survival time of the patients with high miR-21 expression is shorter than that of the patients with low miR-21 expression, indicating its oncogenic role (Fig 3D), which was previously reported [39, 40]. This was shown for miR-155 as well (Fig 3F) [41, 42]. However, the survival of the patients with low miR-34a was shown to be shorter compared with high-miR-34 expression group (Fig 3E), suggesting a tumor suppressive role of miR-34a, which is consistent with an earlier study [44]. The *p*-values of the comparisons between high- and low-expression patient groups, were 0.025, 0.021, and 0.018 for miR-21, miR-34a, and miR-155, respectively.

### Construction and validation of modules

#### Construction of modules using mRNA, protein, and miRNA data

To construct mRNA-sample modules, we first selected 5890 mRNAs with significantly different expression in normal and tumor samples. The SAMBA bi-clustering algorithm was used for the analysis of mRNAs and samples, allowing the duplication of mRNA and sample information in the constructed modules with an overlap factor of 0.1, where 0 indicates full duplication while 1 indicates no duplication. Based on this algorithm, 221 modules, in which mRNAs have similar expression patterns in the samples included in the module, were obtained. Afterward, we filtered out 82 modules with less than 10 mRNAs. The correlation permutation test and a Bonferroni correction were performed 1000 times on 139 modules, and 119 modules with *q*-values less than 0.05 were selected. The modules selected from the permutation test demonstrate that mRNA expression levels are significantly correlated, in contrast with the randomly constructed modules. We expanded the modules by adding the PPI information, and 30 genes were added to each module on average.

Proteins with the average protein-mRNA SCCs in the top 3% in the modules were selected and a subset of proteins was included in each module after the application of the Bayesian network model. Following this, 71 modules containing mRNAs and proteins were constructed. We selected miRNAs with average miRNA-mRNA and miRNA-protein SCC values in top 3% in the modules, and added a miRNA subset into the modules based on the BIC score. Consequently, 52 modules with three types of molecules, mRNAs, proteins, and miRNAs, were constructed (S3 Table). On average, each module contained 69 mRNAs, 10 proteins, and six miRNAs.

#### Validations of three-factor modules

To confirm the functional relevance of the mRNAs and proteins in the constructed modules, we performed the pathway enrichment test using GO, KEGG, and BioCarta data. We showed that at least one pathway is enriched in 84.6% (44/52) of the constructed modules (S4A–S4C Table). To compare the performance of our modules, we constructed two-factor modules without proteins (S5 Table) and at least one pathway was enriched in only 78.7% (59/75) of those modules (S6A–S6C Table).

**Fig 4:**
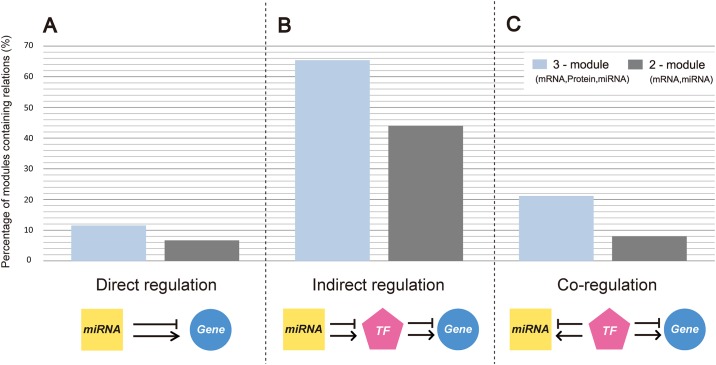
Comparison between three-factor and two-factor modules. (A-C) The percentage of modules containing the information about regulatory relationships. (A) Direct miRNA-gene regulations were analyzed using experimentally obtained data. (B) Indirect regulation was analyzed using experimentally validated miRNA-targeted TFs and genes. (C) Co-regulation was analyzed using the experimentally obtained data, showing TFs targeting miRNAs and genes included in the modules.

We validated miRNA-gene relationships, including both mRNA and protein data (Fig 4). We investigated whether the included miRNAs directly target genes in the same module (S7 Table), and showed (Fig 4A) that at least one miRNA, experimentally shown to target at least three genes in the corresponding module (*q*-value < 0.05), was enriched in 11.5% (6/52) of modules. In contrast to this, in 75 two-factor modules without proteins, at least one miRNA was enriched in only 6.7% (5/75) of modules (S8 Table).

Furthermore, we validated the indirect, TF-mediated, gene regulation by miRNA. We obtained the experimentally validated data about TFs regulated by each miRNA, and their target genes. At least one miRNA that indirectly regulates genes through TFs was found in 65.4% (34/52) of modules (Fig 4B; S9 Table). In the case of two-factor modules without proteins, 44.0% (33/75) of modules were shown to contain at least one miRNA indirectly regulating genes included in the same module (S10 Table).

TF-mediated co-regulation of miRNAs and genes in the same module was validated as well. For each miRNA, we obtained the experimentally validated data about TFs regulating miRNAs and genes. At least one miRNA co-regulated by TFs together with the genes was found in 21.2% (11/52) of modules (Fig 4C; S11 Table). Only 8.0% (6/75) of two-factor modules without proteins were shown to contain miRNAs and mRNAs co-regulated by TFs (S12 Table).

**Fig 5:**
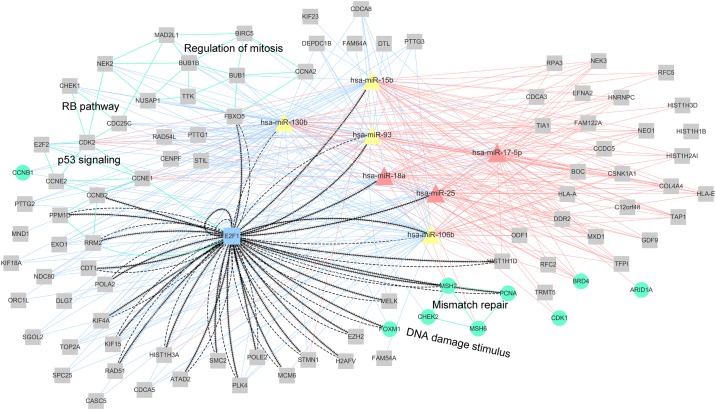
Module 22 network of miRNA, mRNA, and protein interactions. Pink triangles represent miRNAs previously shown to be associated with GBM (HMDD data), while yellow triangles represent the remaining miRNAs that are not included in HMDD or included in HMDD but not shown to be related to GBM. Gray squares represent mRNAs, while blue squares represent TFs. Green circles represent proteins. Pink or blue solid lines represent the negative or positive correlations between miRNAs and mRNAs or proteins, within top or bottom 20%, respectively, of all correlations determined for each miRNA. Dashed lines represent indirect miRNA–TF and TF-mRNA or protein regulations. Lines with contiguous arrows represent the co-regulatory relationships between TFs-mRNAs or proteins and TFs-miRNAs. Green lines indicate that these mRNAs or proteins are related to the same pathways.

A network, included in module 22, containing miRNA, mRNA, and protein interactions and showing some of the enriched pathways is presented in Fig 5. Eleven genes (FBXO5, CDK2, TTK, CDC25C, NUSAP1, BUB1, MAD2L1, NEK2, CCNA2, BUB1B, and BIRC5) were shown to be enriched in mitosis regulation pathway, and three of them (CHEK1, CDC25C, and CDK2) were enriched in RB pathway as well, which plays a central role in the regulation of cancer cell proliferation [45]. Eight genes (CCNB2, CHEK1, RRM2, PPM1D, CCNE2, CDK2, CCNE1, and E2F1) were enriched in p53 signaling pathway, very important for cancer development and progression as well [46]. In this module, E2F1-mediated indirect regulations and two feedback loops between miR-106b and E2F1 and between miR-93 and E2F1 can be observed. Interestingly, these two feedback loops were previously identified as well [47].

**Fig 6:**
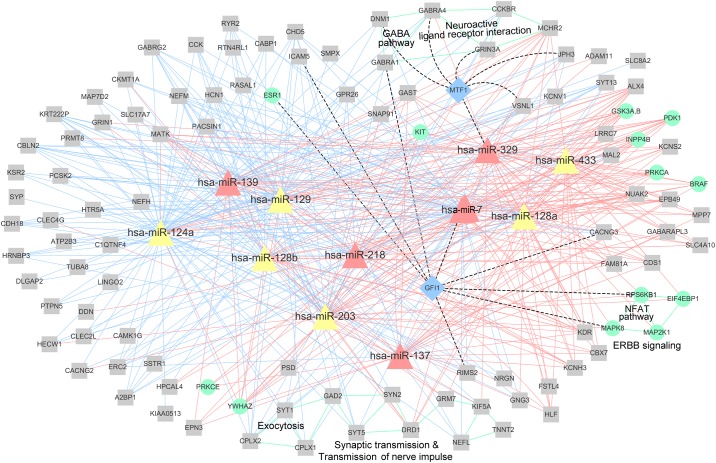
miRNA, mRNA, and protein network in module 50. Pink triangles represent miRNAs previously shown to be associated with GBM (HMDD data), while yellow triangles represent the remaining miRNAs that are not included in HMDD or included in HMDD but not shown to be related to GBM. Gray squares represent mRNAs, while blue squares represent TFs. Green circles represent proteins. Pink or blue solid lines represent the negative or positive correlations between miRNAs and mRNAs or proteins, within top or bottom 20%, respectively, of all correlations determined for each miRNA. Dashed lines represent indirect miRNA–TF and TF-mRNA or protein regulations. Green lines indicate that these mRNAs or proteins are related to the same pathways.

**Fig 7:**
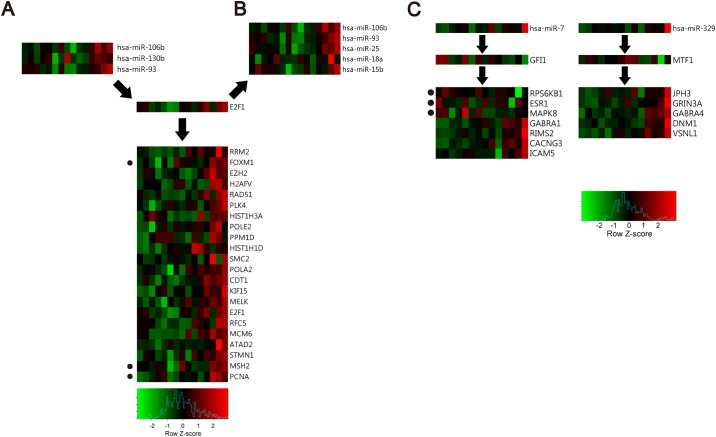
Heatmaps showing the regulation patterns of TF, miRNA, and selected gene and expression levels of the molecules included in modules 22 and 50. (A-C) Red and green boxes show relatively high or low expression levels, respectively, of miRNAs, TFs, mRNAs, and proteins included in these modules, in each sample. The proteins are marked with dots on the left. (A-B) Heatmaps showing indirect TF-mediated regulation of genes, and co-regulation of miRNAs and genes by TFs, in module 22. (C) Heatmaps showing indirect TF-mediated gene regulation in module 50.

A network of miRNAs, mRNAs, and proteins in module 50, together with the enriched pathways is presented in Fig 6. Nine genes (SYT1, SYT5, KIF5A, HTR2A, CPLX1, DRD1, GRM7, SYN2, and GAD2) were enriched in synaptic transmission pathway and transmission pathway of nerve impulse. Three genes (SYT1, CPLX2, and CPLX1) were enriched in exocytosis and five genes (CCKBR, GABRA1, MCHR2, GRIN3A, and GABRA4) in neuroactive ligand receptor interaction pathway, sharing two common genes with GABA pathway (DNM1, GABRA1, and GABRA4), which plays a significant role in glioma cell growth and proliferation in GBM [48, 49]. Furthermore, four genes (MAP2K1, EIF4EBP1, MAPK8, and RPS6KB1) were enriched in ERBB signaling pathway, required for GBM stem cell proliferation [50, 51], and three genes (MAP2K1, MAPK8, and RPS6KB1) were enriched in NFAT pathway, promoting tumor angiogenesis [52, 53]. Module 50 contains directly and indirectly miRNA-targeted genes, and genes and miRNAs co-regulated by TFs. The indirect regulation and co-regulation patterns in module 22 are presented in Fig 7A and 7B, while these regulation patterns in module 50 are presented in Fig 7C.

### Prediction of miR-504 as a tumor suppressor

We further aimed to experimentally demonstrate the role of highly ranked miRNAs in our GBM-related ranking list. Among the highly ranked miRNAs, we selected miRNAs whose role in GBM has not been elucidated yet. Afterward, we examined whether the expression levels of these miRNAs are related to the survival of the patients. MiR-504 was ranked 43*^rd^* and 24*^th^* of 470 miRNAs included in the 5% GBA and 1% GBA ranking lists, respectively, and in our survival analysis it was predicted as a tumor suppressor. However, the role of miR-504 in tumor development and progression has been controversial and it remains unclear [54–57]. In order to elucidate the role of this miRNA further, we experimentally demonstrated that miR-504 acts as a tumor suppressor in GBM.

**Fig 8:**
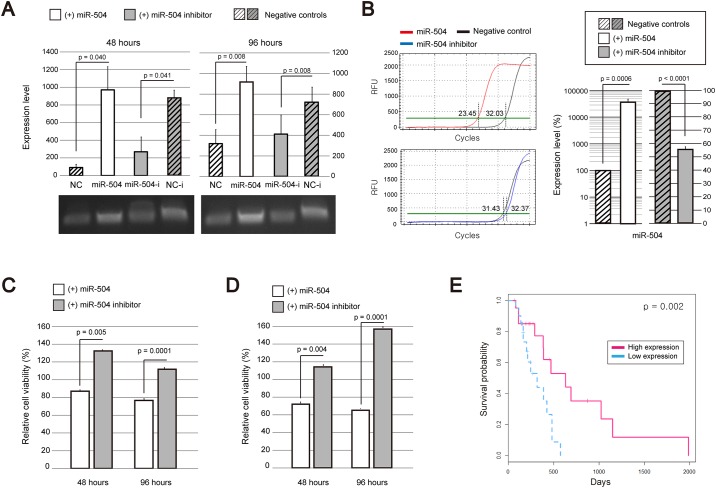
Effects of miR-504 on GBM cell viability and proliferation. (A) Expression levels of miR-504 in non-transfected and transfected cells by miR-504 or miR-504 inhibitor, determined 48 and 96 h after the transfection by RT-PCR. NC, negative control cells not transfected with miR-504; NC-i, negative control cells transfected with miR-504 but without miR-504 inhibitor transfection. The averages of three indented experiments performed in triplicates are presented, with the error bars representing standard errors. Images showing the expression of miR-504 are presented under the plots. *p*-values were calculated using a two-tailed *t*-test. (B) MiR-504 expression levels following the transfection of cells with miR-504 or miR-504 inhibitor determined by q-PCR. The averages of three indented experiments performed in triplicates are presented, with the error bars representing standard errors. The graphs on the left side show the results from one of the experiments. The number of relative fluorescence units (RFU) according to the number of amplification cycles is presented with the thresholds. Red line shows the results of miR-504 transfected cells while the blue line shows the results of miR-504 inhibitor transfected cells. Negative controls are presented with black lines. The number of cycles that meets the amplification threshold for each case is presented. Bar graphs on the right side shows a relative expression level of miR-504 in the cells that are transfected with miR-504 or miR-504 inhibitor compared with that in the negative control cells without transfection. *P*-values were calculated using a two-tailed *t*-test. (C-D) Viability and proliferation rates of GBM cells transfected with miR-504 or miR-504 inhibitor were determined at 48 and 96 h after the transfection. (C) The results of three independent EZ-cytox assays performed in triplicate were averaged, and error bars representing standard error are shown. *P*-values were calculated by using two-tailed *t*-tests. (D) The results of three independent MTT assays performed in triplicate were averaged, and error bars representing standard error are shown. *P*-values were calculated by using two-tailed *t*-tests. (E) The analysis of miR-504 effects on the survival time of GBM patients. Red dotted lines and blue solid lines show overall survival time of GBM patients with high and low miR-504 expression levels, respectively.

We transfected GBM cells with miR-504 and the inhibitor of miR-504, and measured the proliferation rates and viability of the transfected cells in two independent experiments. First, we confirmed that miR-504 is highly expressed in the transfected cells while miR-504 inhibitor transfection represses the miR-504 expression in comparison with the control cells, using RT-PCR (Fig 8A) at 48 and 96 h after the transfection. Following this, miR-504 expression in GBM cells transfected with miR-504 inhibitor was shown to decrease 56.93% on average in comparison with that in the control cells when measured using qPCR (Fig 8B). The proliferation rates and viability of the transfected cells are presented in Fig 8C and 8D, and the obtained results show that the viability and the proliferation rate of GBM cells transfected with miR-504 are significantly decreased compared with those of the cells treated with the miR-504 inhibitor. Furthermore, we confirmed the effects of increased miR-504 expression levels on the survival of GBM patients (*p*=0.002; Fig 8E).

## Discussion

Most studies attempting to predict potential miRNA-gene interactions have used mRNA expression profiles [8–12]. However, mRNA and protein expression profiles of the same genes can differ, as a consequence of different regulatory processes, such as RNA secondary structure, the effects of the regulatory proteins, and codon bias [13–15], which can affect the levels of translation. Therefore, the analyses performed using only mRNA expression data have certain limitations. We confirmed that genes can have different mRNA and protein expression levels, which showed low correlation, with the average SCC of about 0.2. Furthermore, we confirmed that miRNA-gene correlation analyses can be affected by the differences between these two types of data. These results demonstrate the relevance of using protein expression data for gene analyses. We observed that gene expression is regulated at translational level, which is obvious when mRNA-miRNA and protein-miRNA correlations are determined. The results of our study suggest that the rankings generated using both protein and mRNA expression data show better performance in the identification of survival-related miRNAs than the ones generated without protein data. Our results further demonstrated that the three-factor modules including proteins present the direct miRNA regulation of genes better, in comparison with the two-factor modules, which was also observed when TF-mediated indirect regulation and co-regulated processes were examined. This indicates the importance of using both mRNA and protein expression profiles for the identification of cancer-related miRNAs.

**Fig 9:**
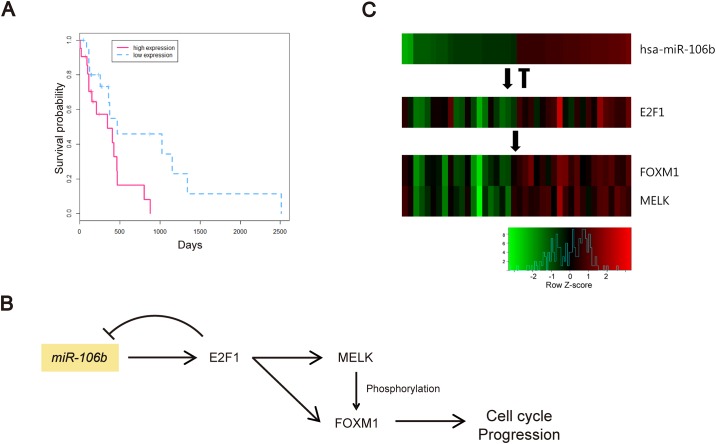
miR-106b-regulated genes in GBM. (A) Survival time of GBM patients depending on miR-106b expression. Red dotted and blue solid lines represent GBM patients with high and low miR-106b expression levels, respectively. (B) The hypothesized model of miR-106b-induced cell cycle progression, through the regulation of E2F1, MELK, and FOXM1. Arrows indicate experimentally validated direct or indirect regulations. (C) Heatmap of miR-106b, E2F1, FOXM1, and MELK expression levels. Red and green indicate high and low expression levels, respectively.

Among the miRNAs experimentally shown to be involved in indirect regulations and co-regulations in the modules, miR-106b was the most frequently identified miRNA. Additionally, miR-106b was shown to be highly ranked (33*^th^* and 8*^th^*, respectively) in 5% GBA and 1% GBA ranking lists. miR-106b was shown to be significantly related to lower survival rates of GBM patients (*p* = 0.010; Fig 9A). In the modules containing miR-106b, we found that miR-106b and E2F1 are mutually regulated, suggesting a feedback loop between miR-106b and E2F1, which was reported in a previous study as well [47]. Among genes predicted to be indirectly targeted by miR-106b via E2F1, FOXM1 and MELK were the most frequently identified genes in several modules. Several previous studies showed that E2F1 promotes the expression of FOXM1 [58], while FOXM1 and MELK form a protein complex, and FOXM1 is activated by phosphorylation [47]. The relationships between miR-106b and MELK or FOXM1 have not been investigated previously, and therefore we hypothesized that this interaction is based on the predicted indirect regulation through E2F1, as shown in Fig 9B. To support the hypothesis, we examined their expression levels, and demonstrated a positive correlation between miR-106b, E2F1, FOXM1, and MELK expression levels (Fig 9C). Since MELK-dependent FOXM1 phosphorylation is necessary for GBM cell proliferation [59], these results may suggest potential therapeutic approaches targeting miR-106b.

**Fig 10:**
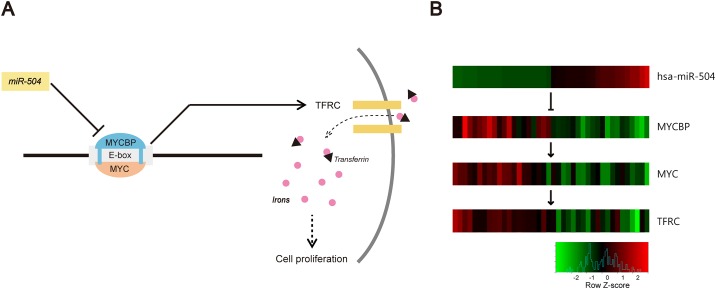
Prediction of miR-504 as a tumor suppressor. (A) The model hypothesizing the mechanisms underlying miR-504 effect on GBM cell proliferation. (B) Heatmap showing the expression levels of miR-504, MYCBP, MYC, and TFRC. Red and green indicate high and low expression, respectively.

In this study, we experimentally confirmed that miR-504 plays a role of a tumor suppressor in GBM. To elucidate the mechanisms underlying miR-504 effects, we identified genes with the expression levels highly related to miR-504 levels. From 146 genes obtained using mRNA and protein expression datasets, we showed that the expression levels of EGFR and TFRC were the ones that showed the highest negative correlations with miR-504 expression levels, and they were included in the same module. However, the relationship between miR-504 and TFRC has not been reported previously. Therefore, we further investigated TFRC, a transferrin receptor controlling the iron uptake in cells, which is required for the proliferation of GBM cells [60, 61]. It was experimentally demonstrated that miR-504 targets MYCBP [62], stimulating c-MYC transcription activity on E-box [63, 64], and TFRC is regulated by c-MYC [65]. This indicates that a potential indirect miR-504 regulation of TFRC, which is mediated by MYCBP and c-MYC, and the expression of these genes is significantly correlated with miR-504 expression. Additionally, they were ranked in top 5% (7*^th^*/146) and 14% (21*^th^*/146), respectively. The hypothesized interactions between these molecules are presented in Fig 10A. We examined the expression changes of these molecules, and heatmap presented in Fig 10B demonstrates negative correlation between miR-504 expression and the expression of these genes.

In future, our approach can be expanded to other cancer types in order to identify cancer-related miRNAs or genes. These analyses can be used for the determination of candidate markers for cancer therapy, following the additional validation by other methods.

## Supporting Information

S1 Table miRNA ranking list constructed using mRNA and protein expression data. MiRNAs are ranked by the influence scores. Ranking by 5% GBA and 1% GBA is presented, together with the information whether the miRNA is included in HMDD.
(XLSX)

S2 Table miRNA ranking list constructed using mRNA expression data alone. (XLSX)

S3 Table mRNAs, proteins, and miRNAs included in three-factor modules. miRNAs, mRNAs, and proteins included in 52 modules constructed with three types of molecules are shown.
(XLSX)

S4 Table Pathway enrichment analyses of genes included in three-factor modules. (A) Enrichment analyses using GO, (B) enrichment analyses using KEGG, and (C) enrichment analyses using BioCarta data.
(XLSX)

S5 Table mRNAs and miRNAs in two-factor modules. mRNAs and miRNAs included in 75 modules constructed with two types of molecules are presented.
(XLSX)

S6 Table Pathway enrichment analyses of genes included in two-factor modules. (A) Enrichment analyses using GO, (B) enrichment analyses using KEGG, and (C) enrichment analyses using BioCarta data.
(XLSX)

S7 Table Direct regulations in three-factor modules. Experimentally validated miRNA-targeted genes are shown.
(XLSX)

S8 Table Direct regulations in two-factor modules. Experimentally validated miRNA-targeted genes are shown.
(XLSX)

S9 Table Indirect regulation in three-factor modules. Experimentally validated miRNA-regulated TFs and TF-regulated genes are presented.
(XLSX)

S10 Table Indirect regulation in two-factor modules. Experimentally validated miRNA-regulated TFs and TF-regulated genes are presented.
(XLSX)

S11 Table Co-regulatory interactions in three-factor modules. Experimentally validated TF-regulated miRNAs and genes are presented.
(XLSX)

S12 Table Co-regulatory interactions in two-factor modules. Experimentally validated TF-regulated miRNAs and genes included in two-factor modules are presented.
(XLSX)
